# Supplementary material for: Transcriptome-Wide Gene Expression Plasticity in Stipa grandis in Response to Grazing Intensity Differences
Source: Int J Mol Sci. 2021 Nov 2;22(21):11882. doi: 10.3390/ijms222111882 (PMC8611654; doi:10.3390/ijms222111882)
Supplement: Supplementary file 1 [file ijms-22-11882-s001.zip › ijms-1386041-supplementary.pdf]

The following are available online at www.mdpi.com/xxx/s1, Figure S1: Correlation analysis of the coefficient of variation filtered transcripts, Figure S2: Gene Ontology functional annotation of the filtered transcripts, Figure S3: Kyoto Encyclopedia of Genes and Genomes annotation of the filtered transcripts

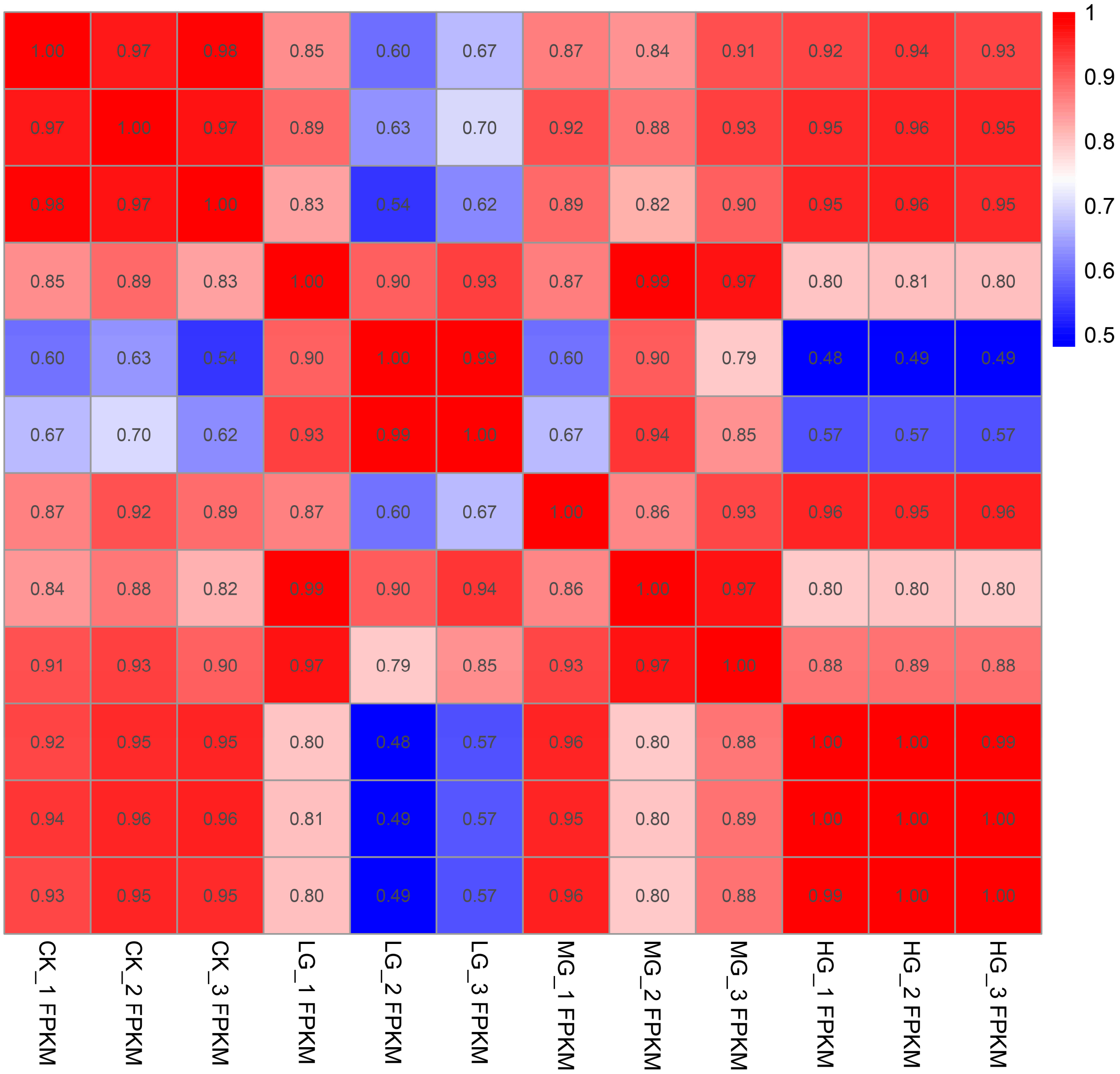

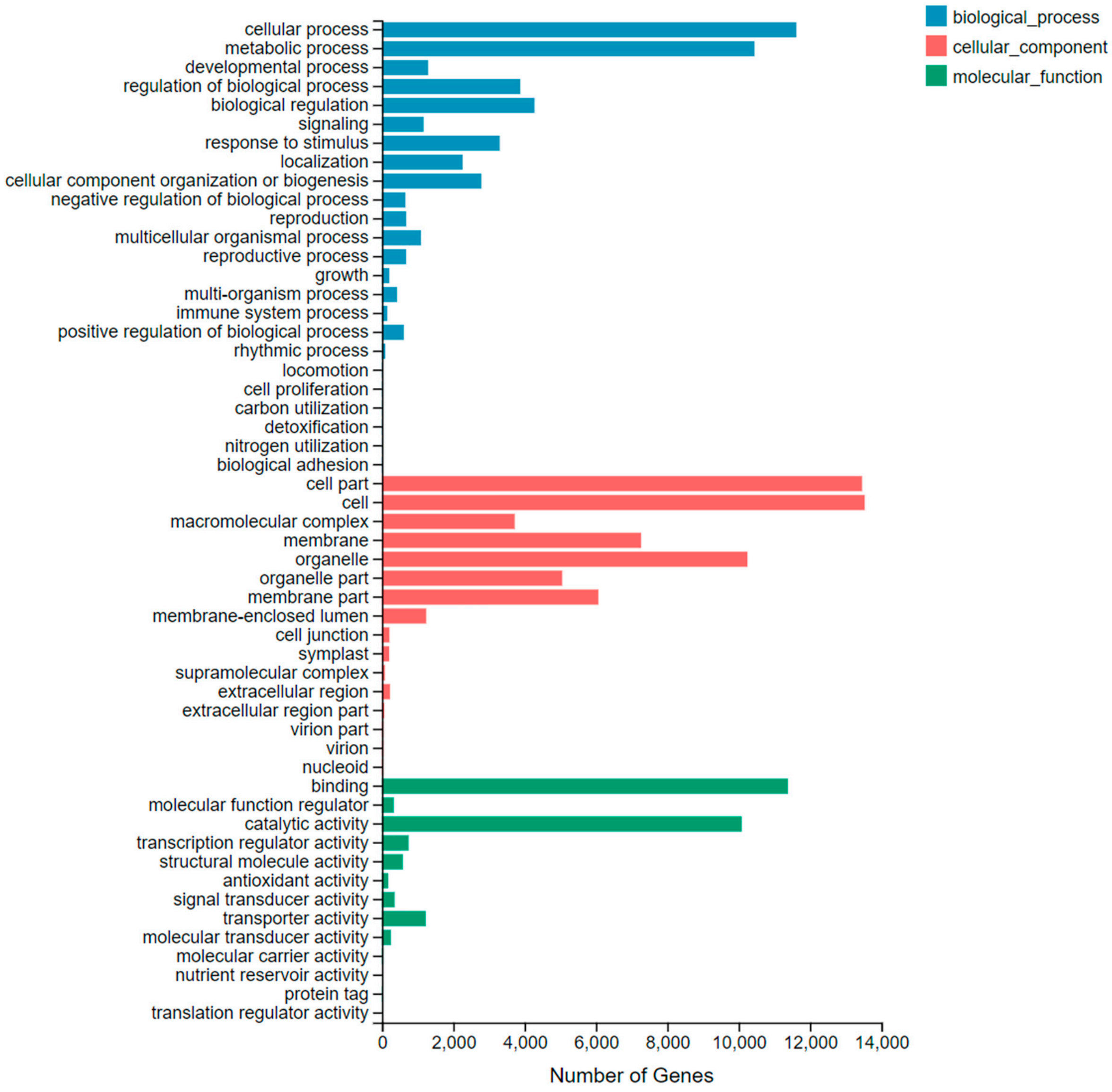

biological_process
cellular_component
molecular_function
cellular process
metabolic process
developmental process
regulation of biological process
biological regulation
signaling
response to stimulus
localization
cellular component organization or biogenesis
negative regulation of biological process
reproduction
multicellular organismal process
reproductive process
growth
multi-organism process
immune system process
positive regulation of biological process
rhythmic process
locomotion
cell proliferation
carbon utilization
detoxification
nitrogen utilization
biological adhesion
cell part
cell
macromolecular complex
membrane
organelle
organelle part
membrane part
membrane-enclosed lumen
cell junction
symplast
supramolecular complex
extracellular region
extracellular region part
virion part
virion
nucleoid
binding
molecular function regulator
catalytic activity
transcription regulator activity
structural molecule activity
antioxidant activity
signal transducer activity
transporter activity
molecular transducer activity
molecular carrier activity
nutrient reservoir activity
protein tag
translation regulator activity
0
2,000
4,000
6,000
8,000
10,000
12,000
14,000
Number of Genes

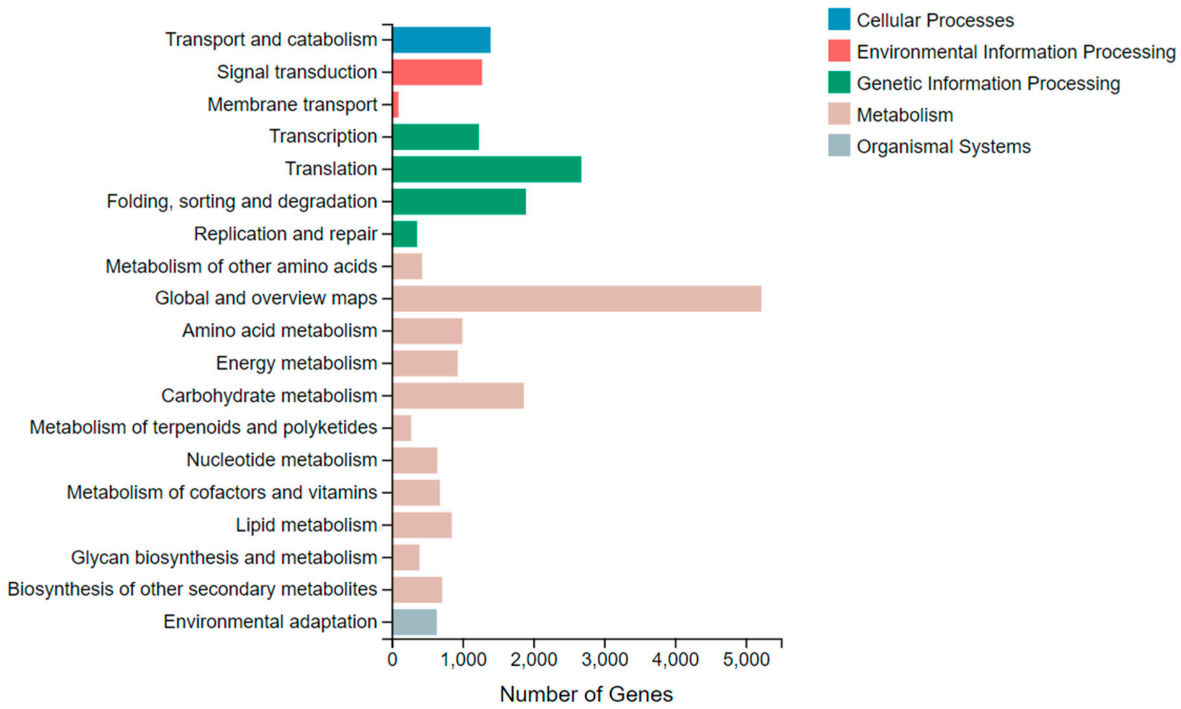

Cellular Processes
Environmental Information Processing
Genetic Information Processing
Metabolism
Organismal Systems
Transport and catabolism
Signal transduction
Membrane transport
Transcription
Translation
Folding, sorting and degradation
Replication and repair
Metabolism of other amino acids
Global and overview maps
Amino acid metabolism
Energy metabolism
Carbohydrate metabolism
Metabolism of terpenoids and polyketides
Nucleotide metabolism
Metabolism of cofactors and vitamins
Lipid metabolism
Glycan biosynthesis and metabolism
Biosynthesis of other secondary metabolites
Environmental adaptation
0
1,000
2,000
3,000
4,000
5,000
Number of Genes
